# Relationship between decreased lower extremity muscle mass and knee pain severity in both the general population and patients with knee osteoarthritis: Findings from the KNHANES V 1-2

**DOI:** 10.1371/journal.pone.0173036

**Published:** 2017-03-15

**Authors:** Yun-Hong Cheon, Hyun-Ok Kim, Young Sun Suh, Min Gyo Kim, Wan-Hee Yoo, Rock Bum Kim, Hyun-Su Yang, Sang-Il Lee, Ki-Soo Park

**Affiliations:** 1 Internal Medicine and Institute of Health Science, Gyeongsang National University School of Medicine, Jinju, Republic of Korea; 2 Department of Rheumatology, Internal Medicine, Chonbuk National University School of Medicine, Chonju, Republic of Korea; 3 Department of Preventive Medicine, Gyeongsang National University School of Medicine, Jinju, Republic of Korea; University of Glasgow, UNITED KINGDOM

## Abstract

**Objective:**

To identify the prevalence of and risk factors for knee pain and radiographic knee osteoarthritis (RKOA) and to investigate the relationship between decreased lower extremity muscle mass (DLEM) and knee pain severity.

**Methods:**

Using data from the Korea National Health and Nutrition Examination Survey, 3,278 participants who were ≥50 years old and who underwent dual x-ray absorptiometry, plain knee radiographs and completed a knee pain questionnaire were enrolled. Lower extremity muscle mass (LEM) was defined as the sum of the fat-free soft tissue mass of the legs, and lower extremity muscle mass index (LMI) was calculated as LEM/body weight (%). DLEM was defined as an LMI more than two standard deviations below the mean of a gender-matched young reference group. Categorical variables were presented as numbers (weighted %).

**Results:**

The prevalence of knee pain and RKOA were 22% (n = 721) and 34.7% (n = 1,234), respectively. Multivariate logistic regression analysis showed being female (OR 2.15, 95% CI 1.67–2.79), older (OR 1.03, 95% CI 1.01–1.04), less educated (OR 1.72, 95% CI 1.09–2.71), stiffness (OR 16.15, 95% CI 12.04–21.66), bed rest (OR 2.49, 95% CI 1.81–3.43), RKOA (OR 2.20, 95% CI 1.78–2.74) and DLEM (OR 1.54, 95% CI 1.09–2.17) were associated with knee pain. Participants with simultaneous RKOA and DLEM complained of more severe pain (pain score 7.18 ± 2.48) than those with knee pain without RKOA or DLEM (5.02 ± 2.44), those with only RKOA (6.29 ± 2.50), or those with only DLEM (6.78 ± 2.18) (P<0.001). These results remained after multivariate analyses of variance (MANOVAs).

**Conclusion:**

The prevalence of knee pain and RKOA were 22% and 34.7%, respectively, in the general Korean population. DLEM was an independent risk factor for knee pain and it was associated with increased pain severity, regardless of RKOA.

## Introduction

The number of elderly people in the world is increasing. With aging, chronic pain, especially knee pain, is becoming a serious social problem, causing impairments in physical function and quality of life, as well as increased morbidity. Although previous studies have revealed multifactorial risk factors for knee pain, including obesity, radiographic changes, female gender, older age, stiffness and previous knee injury, the underlying etiology for knee pain remains poorly understood [1–4]. The prevalence of knee pain differs by country and ethnicity, but approximately 23% to 48% of the elderly population worldwide suffers from knee pain [4–6]. In the case of Korea, only one epidemiologic study has been performed, reporting a 23.1% prevalence of knee pain in the Korean elderly population [1].

Knee osteoarthritis (OA) is the leading cause of productivity loss and long-term disability, particularly in the elderly [4, 7]. Although existing research suggests the prevalence of knee OA varies with sample size, target population, and participant ethnicity, the prevalence of knee pain is consistently shown to increase with age. Knee pain is the main clinical knee OA symptom, which is one of the primary causes of knee replacement surgery [8]. Well-known risk factors for knee pain in patients with knee OA are old age, female gender, and obesity [9]. Another strong risk factor involves radiographic changes. Previous studies have demonstrated that osteophytes [10, 11] and joint space narrowing [12] are closely related to knee pain. In practice, however, physicians are often faced with patients who have no pain, despite receiving grades of 3 or 4 (or higher) for osteophytes and joint space narrowing on the Kellgren-Lawrence (K/L) grading scale. The K/L assesses knee OA severity from a plain radiograph. Recently, accumulating data have suggested that even though radiographic changes have been correlated with knee pain, radiographic change severity is not always proportional to knee pain severity. Studies have suggested that, not only radiographic change, but additional aggravating factors might be related to knee pain severity in patients with osteoarthritis [8, 13].

Body composition changes with aging, including reduced muscle mass or strength and increased fat proportions. Sarcopenia is defined by decreased muscle mass and impaired muscle function; it progresses with aging and is associated with frailty, falls, mortality, and pain risk [14]. In addition, quadriceps or vastus medialis muscle weakness has been shown to be related to knee pain [15, 16]. One cross-sectional study indicated that leg muscle strength is an independent risk factor for knee pain in OA patients [7]. These findings indirectly support the notion that it is not muscle strength or power, but rather lower extremity muscle mass (LEM), that plays an important role in the severity of knee pain. However, little is known about the relationship between knee pain and lower extremity muscle mass itself. In this context, we hypothesized that decreased lower extremity muscle mass (DLEM) is related to knee pain and its severity in both the general population and in patients with radiographic knee OA (RKOA).

The first objective of this study was to identify the prevalence of knee pain, RKOA, and risk factors in the elderly Korean population using data from the fifth Korean National Health and Nutritional Examination Survey (KNHANES V 1–2). Second, we attempted to determine whether DLEM influenced knee pain severity in both the elderly Korean population and in participants with RKOA.

## Materials and methods

### Subjects

**Fig 1:**
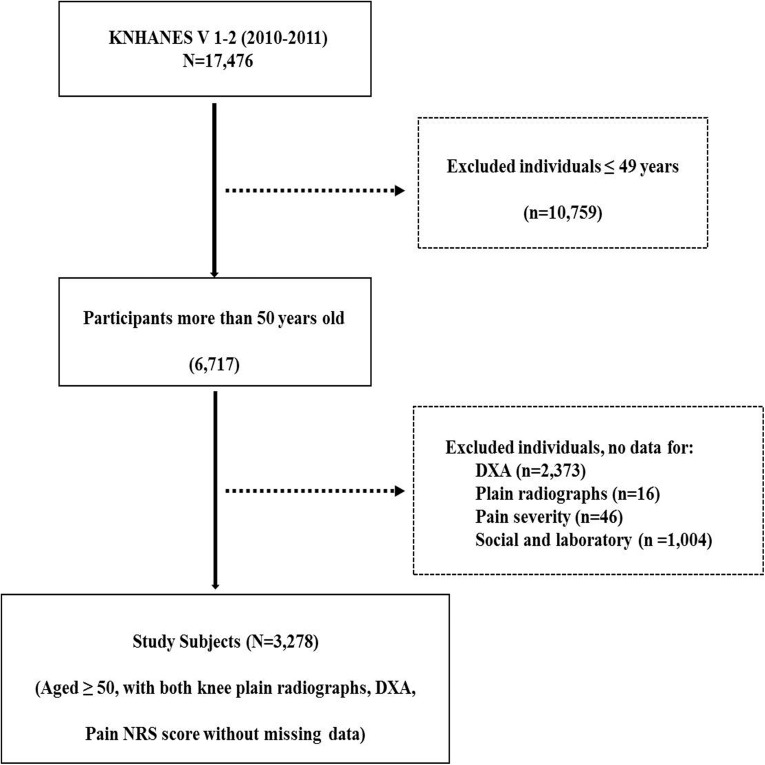
Flow chart. KNHANES; Korea National Health and Nutrition Examination Survey, DXA; dual x-ray absorptionmetry, NRS; numerical rating scale.

The KNHANES is a nationally representative cross-sectional health survey periodically conducted by the Korean Ministry of Health and Welfare since 1998 to assess the health and nutritional status of the Korean population. In Korea, all citizens are covered by health insurance, regardless of income or region, and anyone can be selected to participate in the KNHANES survey. The KNHANES used a stratified, multistage, clustered probability sampling method to select a representative sample of the Korean population. In the case of KNHANES V 1–2, a total of 6,567 households selected from 384 survey regions responded to the questionnaire-based survey and were categorized by region, gender, age, and average household size. The details of the KNHANES V have been previously described elsewhere [1, 17, 18]. This cross-sectional study used data from the fifth KNHANES V 1–2 (N = 17,476; performed from January 2010 to December 2011). Of 17,476 participants, 10,759 (61.6%) were excluded due to age ≤ 49 years. In addition, participants were excluded due to lack of data regarding dual x-ray absorptiometry (DXA; n = 2,373) and knee plain radiographs (n = 16). We also excluded 1,050 individuals due to lack of data regarding knee pain severity, vitamin D level, smoking status, and education level. Ultimately, 3,278 participants (18.8%) were included in this cross-sectional study (Fig 1). All participants provided written informed consent for participation in the survey and for the use of their data for research purposes (IRB 2010-02CON-21-C and 2011-02CON-06-C, obtained by the Korea Centers for Disease Control & Prevention).

### Sociodemographic characteristics

All questionnaires were administered by well-trained government survey staff, primarily consisting of nurses and nutritionists. Smoking status (never, previous, and current smoker), monthly income (low, less than 670 USD dollars a month; moderate, less than 2,200 USD dollars a month; high, more than 2,200 USD dollars a month) and educational level (low, less than elementary school; moderate, middle and high school; high, more than university) were assessed via individual interviews. All participants reported whether or not they experienced bed rest due to any disease in the three months previous to their interview.

### Anthropometric measurements

Body weight and height were measured to the nearest 0.1 kg and 0.1 cm, respectively. Body mass index (BMI) was calculated as weight (kg) / height (m^2^). BMI scores were categorized into three main groups: normal (BMI < 25 kg/m^2^), pre-obese (25-< 30kg/m^2^) and obese (≥ 30 kg/m^2^) according to World Health Organization (WHO) recommendations [19]. However, only 3% of participants were categorized as obese in this study; therefore, we defined obesity as BMI ≥ 25 kg/m^2^, based on criteria for the Asia-Pacific region [20–22]. Waist circumference (WC) was measured to the nearest 0.1 cm at the midpoint between the lower costal margin and the iliac crest during expiration. Smoking status was determined according to whether participants were never smokers, past smokers, or current smokers. Body density was evaluated by DXA (Discovery-W fan-beam densitometer; Hologic Inc., MA, USA), and osteoporosis was defined as a T-score < -2.5. Serum 25(OH) D levels were measured using a gamma counter (1470 Wizard, Perkin-Elmer, Turku, Finland), and vitamin D deficiency was defined as a 25(OH) D level < 20 ng/ml [23].

### Assessment of knee pain and stiffness

Knee pain was defined as the presence of pain in the knee joint lasting more than 30 days during the three months prior to interview. Transient knee pain, which developed within 3–4 days after severe exercise and work, was excluded. Severity of knee pain was evaluated using a 10-point numerical rating scale (NRS; 0, no pain; 10, worst pain ever). Knee stiffness was defined as the presence of morning knee stiffness for more than 30 days during the three months prior to interview.

### Assessment of radiographic changes and definition of radiographic knee osteoarthritis

All participants who were 50 years of age or over were examined with bilateral knee plain radiographs (bilateral anteroposterior and lateral; 30° of flexion, in a weight-bearing anteroposterior position) using a SD 3000 Synchro Stand. Two independent radiologists evaluated radiographic changes related to OA using the K/L grading system [24]. If there was any discrepancy greater than two K/L grades between radiologist scores, a third radiologist was consulted. RKOA was defined as a K/L grade ≥ 2 in both knees.

### Definition of decreased lower extremity muscle mass

DXA allows for identification of body mass components: bone mineral, fat, and fat-free soft tissue. Appendicular skeletal muscle mass (the sum of the fat-free soft tissue mass of skeletal muscle) estimated by DXA is a practical and accurate method for quantifying human skeletal muscle mass [25]. We extracted body composition parameters, including lower extremity fat mass (kg), lower extremity fat percentage (%), and lower extremity muscle mass (kg), with DXA records contained in the KNHANES V 1–2 database. Muscle mass for both legs was calculated from DXA records. Lower extremity muscle mass (LEM) was obtained by subtracting lean mass from the mass of both lower extremities, excluding fat and bone mass. Lower extremity muscle mass index (LMI) was calculated as LEM/body weight (%). Decreased lower extremity muscle mass (DLEM) was defined as an LMI (LEM/weight) value two standard deviations below the mean in a gender-matched young reference group. The young reference group consisted of 1,708 male and 2,278 female participants from the KNHANES V 1–2 who were 20 to 40 years old. DLEM cut-off values were 19.9% for males and 16.2% for females. Finally, to assess the impact of DLEM on pain severity, participants were classified into four groups according to presence of DLEM and RKOA as follows: the reference group (participants with knee pain without DLEM and RKOA), group 1 (participants with knee pain with RKOA and without DLEM), group 2 (participants with knee pain with decreased lower extremity muscle mass and without RKOA), and group 3 (participants with knee pain with both RKOA and DLEM).

### Statistical analyses

Categorical variables are presented as number (weighted %) to produce nationally representative data. Participant clinical characteristics were compared with Student’s *t*-tests for continuous variables and Chi-square tests for categorical variables. Multivariate logistic regression analyses were conducted to evaluate risk factors for knee pain (yes/no) in both the general Korean population and in patients with RKOA. To evaluate the impact of DLEM on pain severity, we compared the pain severity of the four groups using multivariate analysis of variance (MANOVA). A linear trend test was computed to analyze the relationship between pain severity and DLEM. All statistical analyses were performed with statistical software SAS 9.3 (SAS Institute Inc., Cary, NC, USA). A *P* value < 0.05 was regarded as statistically significant.

## Results

### Prevalence of knee pain and its risk factors in the general population

Study participant characteristics are summarized in Table 1. The final sample for analysis in this study included 1,757 females (49.5%) and 1,521 males (50.5%), with a mean age of 61.3±0.24 years. Among participants with knee pain, 12.7% (n = 51) were current smokers (mean duration: 36.7 ± 9.7 years and mean number of cigarettes a day: 16.6 ± 8.1). Mean BMI was 24.0 ± 0.1. The prevalence of knee pain in the Korean population was 22% (n = 721), and the prevalence of RKOA was 34.7% (n = 1,234). Mean age, BMI, WC, prevalence of osteoporosis, stiffness, DLEM, and RKOA were significantly higher in participants with knee pain than in participants without knee pain (*P* < 0.001).

**Table 1:** Characteristics of participants and risk factors for knee pain. ^a^

	Total	Knee Pain	No Pain	*P* Value^*^
**Patient number**	3,278	721	2,577	
**Age (years)**	61.27 ± 0.24	65.14 ± 0.44	60.25 ± 0.23	< 0.001
**Female, n (%)**	1,757 (49.5)	547 (73.4)	1,210 (43.2)	< 0.001
**Height (cm)**	160.75 ± 0.21	156.67 ± 0.38	161.83 ± 0.21	< 0.001
**Weight (kg)**	62.28 ± 0.23	60.31 ± 0.46	62.79 ± 0.26	< 0.001
**BMI (kg/m**^**2**^**), n (%)**				<0.001
< 25	2,140 (64.5)	419 (58.9)	1,721 (66.0)	
≥25 - <30	1,041 (32.6)	251 (33.8)	790 (32.2)	
≥30	97 (2.9)	51 (7.2)	46 (1.7)	
**Obesity (%)**^**†**^	1,138 (34.7)	302 (41.0)	836 (33.9)	< 0.001
**Waist circumference (cm)**	84.02 ± 0.23	84.93 ± 0.45	83.78 ± 0.25	0.014
**Current smoker, n (%)**	556 (21.0)	73 (12.7)	483 (23.2)	< 0.001
**Income status, n (%)**^**‡**^				< 0.001
**Low**	817 (26.0)	217 (30.4)	600 (24.9)	
**Moderate**	1,650 (50.7)	362 (49.2)	1,288 (51.0)	
**High**	811 (23.3)	142 (20.4)	669 (24.1)	
**Education level, n (%)**^**§**^				< 0.001
Low (0–6 years)	1,529 (44.6)	503 (69.2)	1,026 (38.1)	
Moderate (7–12 years)	1,378 (43.7)	188 (26.6)	1,190 (48.2)	
High (≥ 13 years)	371 (11.7)	30 (4.2)	341 (13.6)	
**Stiffness, n (%)**^**||**^	370 (121.3)	297 (41.2)	73 (2.8)	< 0.001
**Vitamin D insufficiency, n (%)**^**¶**^	2,047 (61.4)	457 (62.7)	1,590 (61.1)	0.552
**Osteoporosis, n (%)**	715 (19.5)	244 (31.1)	471 (16.5)	< 0.001
**Bed rest, n (%)**	266 (7.8)	126 (18.0)	140 (5.2)	< 0.001
**Days of bed rest**	0.57 ± 0.07	1.70 ± 0.24	0.28 ± 0.05	< 0.001
**LE fat mass (kg)**	5.00 ± 0.05	5.58 ± 0.10	4.85 ± 0.05	< 0.001
**LE fat percentage (%)**	26.79 ± 0.24	30.78 ± 0.49	25.75 ± 0.24	< 0.001
**LEM (kg)**	13.15 ± 0.08	11.83 ± 0.13	13.50 ± 0.08	< 0.001
**LMI (LEM/weight x 100, %)**	21.03 ± 0.08	11.83 ± 0.13	13.50 ± 0.08	< 0.001
DLEM, n (%)^**^	237 (6.4)	85 (10.7)	152 (5.2)	< 0.001
**K/L grade, n (%)**				< 0.001
Normal	1,210 (39.9)	138 (22.0)	1,072 (44.6)	
K/L grade 1	834 (25.4)	131 (17.0)	703 (27.6)	
K/L grade 2	459 (12.4)	87 (10.2)	372 (12.9)	
K/L grade 3	511 (15.1)	185 (26.2)	326 (12.2)	
K/L grade 4	264 (7.3)	180 (24.6)	84 (2.7)	
**Radiographic knee OA, n (%)**	1,234 (34.7)	452 (61.0)	782 (27.8)	< 0.001

^a^ Categorical variables are presented as number (weighted %), and continuous variables as mean ± SD, unless otherwise indicated.

^*^P values are the results of comparisons between participants with knee pain and participants without knee pain. *P* < 0.05 was considered significant. BMI = Body mass index; DLEM = Decreased lower extremity muscle mass; K/L = Kellgren and Lawrence; LE = Lower extremity; LEM = Lower extremity muscle mass; LMI = Lower extremity muscle mass index; OA = Osteoarthritis.

^†^ Obesity was defined as BMI > 25 kg/m^2^.

^‡^ Income status was categorized into three groups (low, less than 670 USD dollars a month; moderate, less than 2,200 USD dollars a month; high, more than 2,200 USD dollars a month).

^§^ Education level was categorized into three groups (low, less than elementary school; moderate, middle and high school; high, more than university).

^||^ Stiffness was defined as the presence of morning stiffness of the knee for more than 30 days during the past 3 months.

^¶^ Vitamin D insufficiency was defined as serum 25(OH) D level < 20 ng/ml.

** Lower extremity muscle mass and fat mass were evaluated by dual x-ray absorptionmetry. DLEM was defined as an LMI was 2 standard deviations below the mean in a sex-matched young reference group.

The results of multivariate logistic regression analyses showed that female gender (OR 2.15, 95% CI 1.67–2.79), older age (OR 1.03, 95% CI 1.01–1.04), lower level of education (OR 1.72, 95% CI 1.09–2.71), stiffness (OR 16.15, 95% CI 12.04–21.66), and experience with bed rest within the previous month (OR 2.49, 95% CI 1.81–3.43) were significantly related to knee pain. In addition, the prevalence of RKOA (OR 2.20, 95% CI 1.78–2.74) and DLEM (OR 1.54, 95% CI 1.09–2.17) were independently related to increased risk for knee pain (Table 2).

**Table 2:** Multivariate logistic regression analysis: risk factors for knee pain.

	Unadjusted OR	95% CI	P value^*^
**Age**	1.03	1.01–1.04	<0.001
**Female**	2.15	1.67–2.79	<0.001
**Obesity**^**†**^	1.18	0.95–1.46	0.146
**RKOA**	2.20	1.78–2.74	<0.001
**DLEM**^‡^	1.54	1.09–2.17	0.015
**Stiffness**	16.15	12.04–21.66	<0.001
**Osteoporosis**	1.07	0.83–1.38	0.607
**Current smoker**	1.00	0.71–1.39	0.985
**Low income (Ref: high income)** ^**§**^	1.26	0.93–1.69	0.133
**Education (Ref: high education)** ^||^	Ref		
Low	1.72	1.09–2.71	0.021
Moderate	1.07	0.68–1.68	0.784
**Experience of bed rest within a month**	2.49	1.81–3.43	<0.001

Predictors for increased knee pain were examined using multivariate logistic regression analysis.

^*^ *P* < 0.05 was considered significant. DLEM = Decreased lower extremity muscle mass; OR = odds ratio; RKOA = Radiographic knee osteoarthritis.

^†^ Obesity was defined as BMI > 25 kg/m^2^.

^‡^ DLEM was defined as an LMI was below 2 standard deviations from the mean in a sex-matched young reference group.

^§^ Income status was categorized in three groups (low, less than 670 USD dollars a month; moderate, less than 2,200 USD dollars a month; high, more than 2,200 USD dollars a month).

^||^ Education level was categorized in three groups (low, less than elementary school; moderate, middle and high school; high, more than university).

### Knee pain risk factors in participants with radiographic knee OA

Of 3,278 participants, we selected 1,234 participants with RKOA. We subsequently subdivided these into two groups based on the presence of knee pain to investigate the risk factors for symptomatic RKOA. The following variables were significantly related to knee pain in participants with RKOA after adjusting for gender, age, obesity, smoking status, education level, osteoporosis, days of bed rest within the previous month, stiffness, and DLEM (Table 3): age (OR 1.02, 95% CI 1.00–1.04), low income (OR 1.70, 95% CI 1.11–2.58), female gender (OR 2.05, 95% CI 1.41–2.98), days of bed rest within the previous month (OR 2.27, 95% CI 1.40–3.66), low education (OR 2.67, 95% CI 1.18–5.99), and stiffness (OR 15.58, 95% CI 10.37–23.39). In addition, DLEM was also an independent risk factor for knee pain in participants with RKOA after adjusting for other variables (OR 1.71, 95% CI 1.09–2.68, P = 0.020; Table 3).

**Table 3:** Logistic regression analysis for knee pain risk factors in patients with radiographic knee OA (n = 1,234).

	Unadjusted OR	95% CI	*P*	Adjusted OR^*^	95% CI	*P*
**Age**	1.04	1.03–1.06	< 0.001	1.02	1.00–1.04	0.047
**Female**	3.51	2.67–4.63	< 0.001	2.05	1.41–2.98	< 0.001
**Obesity**^**†**^	1.28	1.01–1.62	0.038	1.26	0.94–1.70	0.127
**DLEM**^**‡**^	1.78	1.23–2.58	0.002	1.71	1.09–2.68	0.020
**Bed rest**^**§**^	3.42	2.30–5.08	< 0.001	2.27	1.40–3.66	< 0.001
**Stiffness**	17.87	12.15–26.30	< 0.001	15.58	10.37–23.39	< 0.001
**Osteoporosis**	2.23	1.73–2.88	< 0.001	1.15	0.82–1.63	0.419
**Current smoker**	0.481	0.32–0.72	0.985	0.82	0.49–1.38	0.454
**Low income**	2.01	1.43–2.82	0.133	1.70	1.11–2.58	0.015
**Low Education**	5.04	2.61–9.72	0.021	2.67	1.18–5.99	0.018

Crude odds ratio (ORs) and 95% confidence intervals (95% CI) and ORs from multivariate analysis were determined. *P* < 0.05 was considered significant.

* Adjusted for age, gender, obesity, DLEM, stiffness, osteoporosis, smoking status, income status, education level, and bed rest with logistic regression analysis.

^†^Obesity was defined as BMI ≥ 25 kg/m^2^, based on criteria for the Asia-Pacific region.

^‡^ DLEM (decreased lower extremity muscle mass) was defined as an LMI below 2 standard deviations from the mean in a gender-matched young reference group.

^§^Bed rest was defined as the experience of bed rest due to any disease within the previous three months.

### DLEM amplifies knee pain severity

**Fig 2:**
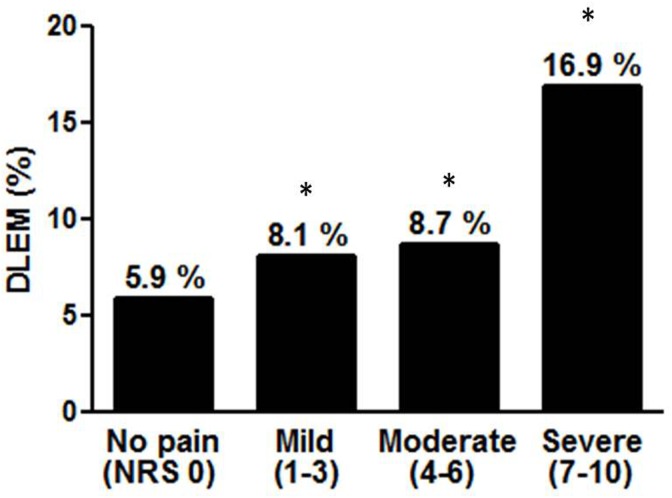
DLEM is positively related with the severity of knee pain. Linear trend test was done to analyze the relationship between pain severity and DLEM. * P value for linear trend < 0.001. DLEM: decreased lower extremity muscle mass; NRS: numerical rating scale.

From the above data, we determined that DLEM was related to knee pain. According to our previous hypothesis, we asked whether DLEM amplified the severity of knee pain in RKOA participants, even though DLEM was not an independent risk factor for knee pain among these participants. Subjects with knee pain (n = 721) were classified into four groups, as described above. Participants with either RKOA (group 1) or DLEM (group 2) complained of more severe pain (NRS, 6.29 ± 2.50 and 6.78 ± 2.16, respectively) than those in the reference group (NRS, 5.02 ± 2.44, P < 0.01). Participants with both DLEM and RKOA (group 3) had significantly more severe knee pain than participants in the reference group or in groups 1 and 2 (Table 4). Multiple regression analyses adjusted for age, gender, education level, smoking status, vitamin D deficiency, osteoporosis, stiffness (model 1), obesity, days of bed rest within the previous month (model 2), and WC (model 3) showed participants in group 3 had more severe knee pain than participants in the reference group, group 1, or group 2 (Table 4). In addition, DLEM was positively correlated with knee pain severity (Fig 2).

**Table 4:** Impact of DLEM on pain severity in patients with or without RKOA (n = 721).

	Number of subjects	Crude		Model 1 ^‡^		Model 2 ^§^		Model 3 ^¶^	
		Pain score	*P*	Pain score	*P*	Pain score	*P*	Pain score	*P*
**Ref**	246	5.02±2.44	-	5.51±2.47	-	5.48±2.48	-	5.49±2.49	-
**Group 1**	390	6.29±2.50	<0.001	5.98±2.38	<0.001	6.00±2.35	<0.001	6.00±2.36	<0.001
**Group 2**	23	6.78±2.16	<0.001	6.86±2.29	<0.001	6.84±2.28	<0.001	6.82±2.28	<0.001
**Group 3**	62	7.18±2.48	<0.001	6.93±2.34	<0.001	6.94±2.37	<0.001	6.88±2.41	<0.001

Values are the mean ± SD for pain NRS scores. Pain severity was evaluated using multivariate analysis of variance (MANOVA). Reference group consisted of participants with knee pain without DLEM and RKOA. Group 1 consisted of participants with knee pain with only RKOA, group 2 consisted of participants with knee pain with only DLEM, and group 3 consisted of participants with knee pain with both RKOA and DLEM. DLEM = Decreased lower extremity muscle mass; RKOA = Radiographic knee OA.

^‡^ Model 1: adjusted for age, gender, education, smoking, vitamin D insufficiency, osteoporosis, stiffness

^§^ Model 2: adjusted for age, gender, education, smoking, vitamin D insufficiency, osteoporosis, current treatment, obesity, days of bed rest

^¶^ Model 3: adjusted for age, gender, education, smoking, vitamin D insufficiency, osteoporosis, current treatment, obesity, days of bed rest, waist circumference

## Discussion

In this study, the prevalence of knee pain and RKOA was 22% and 34.7%, respectively. After adjusting for other variables, DLEM was significantly related to knee pain in both the Korea elderly population and participants with RKOA. DLEM amplified the severity of knee pain and was also positively associated with knee pain severity.

This finding on the estimated knee pain prevalence rate in the general Korean population is comparable with findings from previous studies in US (22.9%) [2] and Swedish populations (25.1%) [6]. However, the rate herein is lower than observed rates in previous studies from the UK (36%) [26] and Vietnam (48.5%) [5]. The present study was restricted to participants aged 50 years and over, was based on DXA and plain radiographs, and evaluated the prevalence of knee pain using the single question “Have you experienced pain in either of your knee joints lasting more than 30 days during the last 3 months?” Unlike our study, the previous UK study targeted participants aged 65 years and over and evaluated knee pain during the previous 12 months [26]. The Vietnam study targeted patients over 40 years of age and evaluated knee pain during the past 12 months [5]. Accordingly, these differences in findings on the prevalence rate of knee pain are likely due to population characteristics and varying definitions of knee pain.

Previous studies demonstrated that aging, female gender, obesity, radiographic changes, older age, stiffness and lower economic status were related to knee pain [1–4]. Consistent with previous studies, we also found that participants who were older, female, with lower economic status and lower education levels reported greater knee pain severity. In the case of female gender, many studies found that females have high risk factors for knee pain and/or knee OA. Silverwood V et al. published an excellent systematic review and meta-analysis. In that study, they reviewed 11 cohort studies from the UK, USA, and Japan, which revealed female gender was a potential risk factor for knee OA and pain [3]. In addition, another study revealed the prevalence of knee pain among non-Hispanic white and Mexican American participants using data from the National Health and Nutrition Examination Survey. Knee pain was was 26.1% for females and 19.7% for males, after adjustment for weight, age, and BMI. In addition, the prevalence of knee pain in the Framingham Osteoarthritis Study was 32.9% for females and 27.7% for males [2]. The reason why women tend to have more knee pain than men has not been confirmed; however, the high prevalence of patellofemoral pain syndrome, lower extremity muscle strength, and anatomical alignment factors were suggested as possible explanations [27].

Socioeconomic characteristics such as income and education play a significant role in the severity of knee pain [1, 3, 4]. A number of previous studies have demonstrated that lower socioeconomic status, including low education and income, is associated with the prevalence of knee pain. One recent study found that socioeconomic inequality was related to frequent knee pain in a large population-based cohort of middle-aged and elderly people in southern Sweden [28]. They suggested that individuals with lower socioeconomic status have lower self-efficacy and use more passive and less effective coping strategies for knee pain. In the current study, we also found that participants in both the general and RKOA groups who had a lower income and less education suffered from severe knee pain, as seen in previous studies. These results suggest that control of knee pain can be achieved not only through exercise and medication, but that public services such as education are also important. Further studies are needed to assess the effects of education on the severity of knee pain.

In the present study, current smoking was negatively associated with knee pain severity in a univariate analysis, although it did not remain significant in a multivariate analysis. Previously, only a few studies have found an association between smoking and knee OA or knee pain; however, these results were controversial and the causal pathway between smoking and knee pain was still unclear. Some studies have reported that smoking was positively associated with knee OA or pain in the presence of greater cartilage loss [29], while others have suggested a possible protective effect of smoking on knee OA [30]. Felson et al. showed that severe OA was less frequent in heavy smokers compared to nonsmokers using data from the 1^st^ Health and Nutrition Examination Survey, even though the possible mechanisms were still unknown [31]. This result was consistent with our data. Because this study was cross-sectional, we were unable to evaluate possible causal pathways between smoking and knee pain. Further, well designed randomized controlled studies will be needed to demonstrate the true relationship between smoking and knee pain.

Obesity is another well-known risk factor for knee OA and knee pain [32.^33^]. The work of Frilander H. et al. demonstrated that obesity, even at the age of 20 years, was associated with knee pain [33]. However, the results were somewhat controversial, and varied by participant ethnicity and other included variables. One population-based cohort study including more than 5,000 participants showed that high BMI was not associated with knee pain [34]. In our study, obesity was not related to knee pain in either the general population or RKOA participants. These different results might be derived from different criteria for obesity. Most previous studies defined obesity as BMI ≥ 30 kg/m^2^, which is typically accepted in Western countries. However, in this study, we defined obesity as BMI ≥ 25 kg/m^2^, because the mean BMI of our participants was 24.04 ± 0.06 kg/m^2^ (participants with knee pain, 24.54 ± 0.16 kg/m^2^ vs participants without knee pain, 23.91 ± 0.07 kg/m^2^) and only 3% of patients had a BMI ≥ 30 kg/m^2^. It is well known that Asian people are usually less obese than Western people, therefore, many Asian countries, including Korea [22], Japan [20], and Taiwan [21], use a BMI of 25 kg/m^2^ as the cut-off value for obesity. Consistent with our study, one study demonstrated that a BMI ≥ 25 kg/m^2^ was not related to knee pain after controlling for age and gender [35]. In addition, a Japanese study also used BMI ≥ 25 kg/m^2^ when they investigated the association between radiographic knee OA and knee pain [36]. Therefore, we speculated that using BMI ≥ 25 kg/m^2^ as a cut-off point for obesity in this study was suitable; this different criterion for obesity may explain why obesity was not related to knee pain in the current research, unlike in findings from previous studies.

Stiffness was the factor most strongly related to knee pain in this study. This result was consistent with previous studies. Schiphof et al., reporting on data from the Rotterdam population-based cohort study, showed that subjects with stiffness had a higher risk for knee pain (OR 1.88, 95% CI 1.48–2.39) in [34]. They speculated that stiffness was correlated with OA prevalence, because stiffness is an OA symptom. Another study revealed that the prevalence of knee stiffness was associated with reduced muscle mass [17]. The cause-effect relationship between stiffness and knee pain was unclear, and we were unable to evaluate causality, as our study was cross-sectional. A well-designed prospective study will be needed to determine the cause-effect relationship between knee stiffness and knee pain.

Several studies have investigated the relationship between thigh muscle mass and knee pain [15, 16, 37, 38]. Those studies evaluated muscle mass using computed tomography and magnetic resonance imaging. However, those methods are time and cost consuming and relatively difficult for primary physicians to use. Recently, a few studies calculated appendicular skeletal muscle mass (the sum of the fat-free soft tissue mass of skeletal muscle) by DXA and suggested that DXA was a practical and accurate method for quantifying human skeletal muscle mass [17, 25]. In addition, many studies have defined reduced body muscle mass, or sarcopenia, as the skeletal mass index two standard deviations below the mean in gender-matched young reference groups (whole body appendicular skeletal muscle mass/body weight [%]) [39]. In this study, we focused only on the lower extremities; therefore, we modified the concept of skeletal mass, and created a lower extremity muscle index as described in the materials and method section. This is the first study to use the lower extremity muscle mass index based on DXA.

Recently, one study investigated the relationship between lower leg muscle mass and knee pain using DXA [17]. Consistent with this study, they found that relatively low leg muscle mass compared to whole body muscle mass was associated with knee pain in participants with radiographic knee OA using data from fifth KNHANES V 1–2. However, there were several differences relative to our study. First, while the current study used cut-off values for DLME derived from a gender-matched young reference group, the other study evaluated participant lower leg muscle mass by calculating the ratio of leg to whole body muscle mass, but not relative to a reference group; therefore, it was difficult to determine a robust cut-off value for lower leg muscle mass. Both studies were cross-sectional, and used data from the fifth Korean National Health and Nutritional Examination Survey. Therefore, we thought calculating a cut-off value from a reference group was important. Second, the previous study enrolled participants aged 50 years or older with RKOA who underwent both radiographs and DXA scans; participants without RKOA were excluded. In the current study, we enrolled participants with or without RKOA and/or knee pain to determine the risk factors for knee pain in both the general and radiographic knee OA populations. Third, unlike the previous study, we analyzed the relationship between DLEM and knee pain by adjusting for other possible risk factors, including income status, education status, smoking status, vitamin D level, and osteoporosis.

Previous studies have revealed a discrepancy between radiographic changes and knee pain in patients with RKOA. Numerous factors, such as age, morning knee stiffness, high BMI, temporal patterns of knee pain, depression, and anxiety, have been proposed to explain this discordance [4, 34]. However, the causes remain unknown. Throughout this study, we found that there was a modest association between knee pain and DLEM. Consequently, we hypothesized that the presence of DLEM explained this discrepancy between radiographic changes and pain in patients with RKOA. To evaluate this hypothesis, we divided participants with knee pain into four subgroups depending on the prevalence of RKOA or DLEM to determine the possible role of lower extremity muscle mass in knee pain severity. We found that participants with DLEM had more severe knee pain than those with normal lower limb muscle mass. In addition, following adjustment for age, gender, obesity, WC, and days of bed rest within the month participants with concurrent DLEM and RKOA were shown to suffer from more severe knee pain compared to participants with only RKOA. These results suggest that DLEM amplified pain severity in patients with knee pain, regardless of the presence of radiographic knee OA. Therefore, we suggest that DLEM is one explanation for knee pain aggravation.

There are several hypotheses regarding the relationship between DLEM and the knee pain severity. First, muscle mass is a major determinant of muscle strength and is important for pressure distribution throughout the joint [40]. Therefore, DLEM may aggravate knee pain via muscle weakness which diminishes the ability to disperse load across the joint, and decreases shock absorption [41]. Second, altered proprioception (awareness of joint position and movement) is also related to knee pain. Decreased lower extremity muscle mass (DLEM) can impair muscle mechanoreceptors, thereby decreasing proprioceptive acuity [38, 41]. Third, when muscle mass is reduced, pro-inflammatory cytokines, including tumor necrosis factor-α, interleukin (IL)-1, and IL-6, can induce the breakdown of myofibrillar proteins. This chronic low-grade inflammatory state can increase peripheral sensitization of nociceptive afferent neurons in muscles and central sensitization, leading to a stronger pain response [42, 43].

This study has several limitations. First, we did not evaluate longitudinal knee pain patterns or the cause-effect relationship between knee pain and risk factors, due to the cross-sectional nature of this study. Second, there was no personal medication information in the KNHANES survey questionnaire; therefore, we could not exclude the direct effects of analgesics, such as non-steroid anti-inflammatory drugs, which are highly correlated with pain levels. Third, it is well known that scores based on the Western Ontario and McMaster Universities Osteoarthritis Index (WOMAC)—rather than an NRS scale—are more appropriate for pain assessment in patients with OA. In the KNHANES V1-2 survey, however, pain was assessed with an NRS scale. Accordingly, we were unable to determine the relationship between DLEM and functional status based on the KNHANES V1-2 dataset. Forth, this study was not able to analyze the effects of potential risk factors such as depression, anxiety, knee alignment, edema, or ligament injury in elderly patients because those factors were not surveyed in the KNHANES questionnaire.

In conclusion, the prevalence of knee pain and RKOA were 22% and 34.7%, respectively, in the general Korean population. Decreased lower extremity muscle mass was an independent risk factor for knee pain in both the general population and in participants with knee OA. In addition, DLEM was positively related to pain severity. Therefore, we suggest that DLEM might be an independent reason for knee pain in the general population and also in subjects with knee radiographic changes. Further studies are needed to identify the role of DLEM in knee pain.
